# Patient and Healthcare Worker Perspectives on the Integration of Mental Health Services Into Routine ART Services in Windhoek, Namibia: A Qualitative Study

**DOI:** 10.5334/ijic.8951

**Published:** 2025-05-29

**Authors:** Ndeshiteelela Kaleinasho Conteh, Ozayr Mahomed

**Affiliations:** 1 University of KwaZulu-Natal, ZA

**Keywords:** Mental health, Mental health integration, Namibia, PLHIV

## Abstract

**Purpose:**

Mental health illnesses are more common in people living with HIV (PLHIV) than in the general population, and mental health issues can make it more difficult for individuals to stay engaged in HIV care, which can negatively affect treatment outcomes. Therefore, integrating mental health services into routine HIV care can improve treatment outcomes for PLHIV. This study assessed patients’ and healthcare workers’ perceptions of enablers and barriers to integrating mental health interventions in routine Antiretroviral treatment (ART) services in Windhoek, Namibia.

**Design/Methodology:**

In-depth interviews were conducted to explore the perspectives of PLHIV, healthcare providers providing ART services, and health managers supporting the national HIV program on integrated mental health services. To assist with data organization, data analysis software (NVivo V.12) was used. Colaizzi’s (1978) inductive thematic analysis was then applied to explore key concepts.

**Findings:**

The Barriers reported were the lack of awareness of mental illnesses among PLHIV, healthcare worker attitudes, limited physical space at health facilities, insufficient financial resources to address staff shortages, and lack of training for healthcare workers. The enablers reported were integrated screening of mental illnesses, training of Healthcare workers, management support, integration policies and guidelines, and community sensitization and awareness on mental health issues.

## Introduction

Globally, an estimated 39 million people were living with HIV at the end of 2022 [1] with Sub-Saharan Africa having a burden of over 70% of global HIV/AIDS cases. Although HIV infection rates in some African countries have plateaued, countries like South Africa 23%, Nigeria 15%, Namibia 12.6%, Uganda 10%, Mozambique 8%, and Kenya 7% have shown persistent high prevalences [2,3]. Mental health illnesses are more prevalent in PLHIV than in the general population [4,5,6]. Results from a systematic study conducted in the United Kingdom (UK) reported a prevalence of depression ranging from 17–47% among PLHIV compared to 2–5% of the UK general population [7]. Similarly, a systematic review study conducted in the United States of America reported a prevalence of depression in women who are HIV positive to be four times more than HIV seronegative women [8]. Literature findings in Africa have reported a similar trend, indicating a higher prevalence of mental illness among PLHIV compared to the general population. About 26–38% of PLWH in South Africa have a mental disorder compared with 13% in the general population [9], which highlights a greater burden on mental illness in PLHIV in comparison to the general population.

There is a variation in mental illness prevalence among PLHIV in African countries. A cross-sectional study conducted at the Cape Coast in Ghana among 391 PLHIV indicated a prevalence of depression, anxiety, and stress to be at 28.6%, 40.8%, and 10.6%, respectively [10]. Another cross-sectional study conducted in Cote d’Ivoire and Senegal among older PLHIV reported a prevalence of depression to be 17.9% [11]. This trend is similar to a cross-sectional survey conducted in Abuja, Nigeria, that found a prevalence of depression to be 28.2% among PLHIV [12], 2.8% for suicide ideation, and 7.8% for alcohol use disorder [12]. A cross-sectional study that was conducted in Zambia among 185 PLHIV found that 17.9% of patients were positive for a mental disorder [13]. Similarly, a cross-sectional study conducted in Cape Town, South Africa, among 65 PLHIV found that 56% of patients had at least one mental disorder at baseline and 48% of patients had at least one disorder at 6months [14]. This evidence indicates that mental disorders are prevalent among PLHIV, highlighting the intersection of HIV and mental illness.

There are multiple factors contributing to the high co-morbidity of HIV/AIDS and mental health illnesses. Factors such as poverty, low education, unstable housing, and food insecurity are noted to contribute to increased vulnerability to HIV infection [15] and also developing mental illness. For example, findings from a cross-sectional study conducted in PLHIV in Jimma Zone, South West Ethiopia, that assessed the association between mental illness and food security, found a prevalence of severe household food insecurity in PLHIV with mental disorders to be 38.7 % [16]. Another cross-sectional study conducted in Fann University Hospital in Senegal and the Treichville and Yopougon Attie hospital in Cote d’Ivoire reported that PLHIV with severe depressive symptoms are likely to be unemployed [11].

Mental health illnesses can make it harder for people to stay engaged in HIV care and, therefore, negatively impact treatment outcomes. Research shows that individuals with mental illnesses are less likely to achieve viral load suppression. A retrospective analysis study among PLHIV conducted in the United States during the period 2008–2020 found that PLHIV that had mental illness were less likely to achieve viral load suppression than those without a mental disorder [17]. Various studies have linked mental illness, specifically depression, to be a strong predictor of poor ART adherence [18]. A comprehensive meta-analysis discovered a notable link between depression and non-adherence to antiretroviral therapy (ART) across 95 distinct samples. It indicated that individuals experiencing depressive symptoms were 42% less likely to achieve optimal (80%) adherence to ART compared to those without such symptoms across 111 separate samples [18]. Another review and meta-analysis, drawing upon data from 125 studies encompassing 19,016 patients across 38 countries, shed light on the multifaceted challenges to adherence. It identified self-reported depression, alcohol consumption, and substance misuse as significant barriers among the top 15 factors hindering treatment adherence. This synthesis underscores the complex interplay of psychological, behavioral, and situational factors impacting patients’ ability to adhere to prescribed treatments effectively [19]. Additionally, various literature has associated increased viremia with different mental health illnesses [20,21] emphasizing the prevalent intersectional vulnerabilities between mental illness and HIV [22].

The World Health Organization (WHO) and the Joint United Nations program on HIV/AIDS (UNAIDS) have emphasized the need for HIV and mental health integration by launching targets and tools to support and facilitate integration [22,23]. Globally, there are models utilized to integrate mental health care into HIV services to 1) increase mental health screening and treatment in ART clinics, 2) incorporate HIV care into mental health clinics, and 3) develop specific sub-speciality clinics serving persons with HIV and mental illness, and have shown positive patient outcomes [24,25,26]. Integration of mental health care into HIV services can be done at micro, meso, and micro level [6]. Micro-level integration focuses on integration at the clinical level, whereby patient-centred care is coordinated in a single process. The integration at the micro level merges a patient’s care in HIV and mental health into one plan [6], while meso-level integration involves professional and organizational integration. Meso-level integration entails collaborative networks and relationships between institutions providing HIV, mental health, and/or substance abuse services with shared responsibilities and accountability between partners [6]. Macro level integration is much higher level than meso and micro level integration, where HIV, mental health, and primary health care sector’s delivery systems are integrated in providing care.

Namibia has made great progress towards responding to the HIV and AIDS epidemic and attaining the UNAIDS 90-90-90 goals. In 2017, the adult HIV prevalence was at 12.6% [27], with 86% of PLHIV knowing their status, 96% of known HIV positives were on treatment, and 91% of those on treatment are virally suppressed [28]. One of the ways to sustain the gains achieved with epidemic control in Namibia is to address leading co-morbidities in PLHIV, such as mental illnesses that have the propensity to impact HIV prevention and treatment efforts negatively. Integrating mental health screening and care into all HIV testing and treatment settings would boost the results of HIV prevention and care [15].

However, the integration and decentralization of mental health services is non-existent at the primary health care level in Namibia [29]. Currently, HIV/AIDS services and mental health services in Namibia are offered separately. HIV care is integrated at the Primary Health Care (PHC) level [30] while mental health services are concentrated at the curative level at the 2 psychiatric hospitals in Namibia. Data from WHO shows that only two health facilities offered outpatient mental health services in 2020 [31]. Namibia has 309 health centers, 34 district hospitals, and 4 intermediate and referral hospitals [32]. Moreover, there is minimal evidence about professionals’ perceptions regarding integrated care [33,34,35], and their perceptions are important in delivering quality care and successfully integrating mental health services into primary care. Patient perspectives of the health care system are equally critical in measuring the quality of health care systems as patient perspectives can provide essential and holistic information on the overall quality of health care [36,37]. This study assessed patients’ and healthcare workers’ perceptions of enablers and barriers to integrating mental health interventions in routine ART services.

## Methods

### Study Area and Study Setting

This study was conducted in Windhoek, Namibia’s capital. Windhoek has 12 public health facilities providing ART services to PLHIVs. In 2022, the Ministry of Health and Social Services (MOHSS) database reported 1703 adult PLHIVs active on ART from August to October 2022.

### Study design

A phenomenological approach [38] was used to explore the perspectives of PLHIV, healthcare providers, and health managers on integrating mental health services into routine HIV programs through in-depth interviews with 20 participants. Understanding these perspectives is valuable for successful integration and improving treatment outcomes. This design allowed researchers to identify perceived barriers and enablers of integrating mental health services at the PHC level.

### Recruitment of study participants

Six public health facilities were purposefully chosen based on the high influx of patients on ART. For patients to be eligible, they had to be 18 years old and older and receive ART services at a public health facility. Healthcare workers and health managers were recruited in the study using the snowball sampling through the referrals from MOHSS nurse mentors. No participant refused enrolment or dropped out of the study. The healthcare workers were eligible to be enrolled in the study if they provided clinical care to patients on ART in public health facilities in Windhoek. Health managers were eligible to be enrolled in the study if they provided technical support to the national HIV program.

The required sample size for the study was determined by considering the findings from prior research, the advice from various research books, and the specific phenomena being investigated. The literature suggests that an appropriate sample size for a phenomenological study can range from 3 to 25 participants [39]. However, this study followed the data saturation principle, as saturation is the most essential element of data adequacy in qualitative research [40]. This principle ensures that both the quantity (detailed description) and quality (comprehensive description) of the data are sufficient. In-depth interviews were conducted in person with study participants.

### Data collection and procedure

Data collection occurred in September 2022 in Windhoek, Namibia. The semi-structured interview guide for healthcare workers included questions to gauge their understanding of mental health and its causes, as well as their opinions on enablers, barriers to mental health integration, and recommendations. The patient interview guide focused on assessing patients’ understanding of mental illness, its management in the community, enablers and barriers to reporting mental illness, and their recommendations. The data collection tool was piloted before data collection to improve validity and feasibility.

The principal investigator (PI) and the health assistants who spoke English and Afrikaans conducted the interviews. English and Afrikaans are commonly spoken languages in the capital city, Windhoek. Probing was applied, when necessary, to get further clarifications from the participants. For the patients and healthcare workers, interviews were conducted in a private room at the health facility to ensure privacy and confidentiality. Health managers were interviewed in their offices at a time that was convenient for them. Achieving concept saturation helped establish how many interviews were necessary. There were no follow-up interviews.

### Trustworthiness of the study

Although the lead researcher and the research assistants speak Afrikaans and English with fluency, all participants expressed that they wanted to be interviewed in English. The principal investigator and research assistant have prior research expertise in the health sector and are knowledgeable about HIV service delivery in Namibia. Peer debriefing with a mentor was done to practice reflexivity. Verbal consent was provided by participants to take part in the research and have their interviews recorded. The health care providers and health managers took an average of 45 minutes, and patients took an average of 30 minutes. Interviews were transcribed verbatim, and the lead researcher checked the accuracy of the transcriptions by comparing them to the audio. By using this method, the findings’ credibility was increased.

### Data Analysis

All data was transcribed in English, and there was no need for translation. Transcribed scripts were coded using NVIVO 12 using thematic analysis by 2 different coders. Transcripts were de-identified and were not returned to participants. We employed deductive and inductive methods to account for categories from the data and those known a priori. The hybrid approach of using inductive and deductive analysis aids with improving the rigor of the analysis approach [41] while also allowing the coder to add emerging themes from data analysis guided by the seven-step phenomenological analysis framework and the Colaizzi (1978) inductive thematic analysis approach [38]. This study applied six out of seven steps of the phenomenological analysis framework: Step 1 of the phenomenological framework involved the reading and familiarization with the transcripts, Step 2 was about identifying key statements that are related to the research question, Step 3 involves careful clustering of themes that are common across all accounts, Step 5 involved the full description of all themes identified in step 4, Step 6 was were a structure of the phenomenon was done by outlining short and dense statements to capture the necessary aspects of the study, Step 7 involved the verification of the fundamental structure if necessary [38]. This step was not performed in this study. The results of this study are presented with the use of thematic headings and quotes.

### Ethics and Informed Consent

Ethical approval for the study was granted by the University of Kwazulu-Natal’s Biomedical Research and Ethics Committee (Ref: BREC/00002904/2021) as well as the Ministry of Health and Social Services’ ethical committee (Ref: 17/3/NKC). The principles of the Helsinki Declaration of 1964 were upheld during the study. Study respondents gave verbal consent to participate in the study as well as to have the interviews recorded. Also, participants were given the choice to withdraw from the study at any point if they wished to. No personal identification information like first and last name, identity numbers was collected to ensure the anonymity of participants. All data was transferred daily from the recorder to a password-protected computer and then deleted from the recorder. Only the Principal Investigator (PI) had access to the password-locked computer.

### Patient and public involvement

None

## Results

### Characteristics of study participants

A total of 20 in-depth interviews were conducted. Of the 20 participants, 10 were PLHIV, 5 were healthcare workers providing HIV services, and 5 were health managers responsible for providing technical support to the national HIV program. Overall, for patient participants, there was an equal representation of participants by sex. Additionally, 4 participants (40%) were aged 22–30 years, and 8 PLHIV (80%) reported that they were single. A total of 5 healthcare workers were interviewed. Three of the participants were under the age of 40 years and were mostly females [4]. Most healthcare workers (80%) worked at a clinic, of which 3 of the 5 are registered nurses. A total of 3 (60%) health managers were between the ages of 34–40 years. Most of the Healthcare worker participants were females (60%) and nurse mentors (Table 1).

**Table 1:** Demographic characteristics of participants.


	CHARACTERISTICS	FREQUENCY (N)	PERCENT%

**Patients (n = 10)**	**AGE**		

	22–30	4	40

	30–40	3	30

	40–50	3	30

	**SEX**		

	Female	5	50

	Male	5	50

	**MARITAL STATUS**		

	Single	8	80

	Married	2	20

**Healthcare workers (n = 5)**	**AGE**		

	28–40	3	60

	41–51	2	40

	**SEX**		

	Male	1	20

	Female	4	80

	**ROLE**		

	Registered Nurse	3	60

	Medical Doctor	1	20

	Health Assistant	1	20

**Health Managers (n = 5)**	**AGE**		

	34–40	3	60

	41–42	2	40

	**SEX**		

	Male	2	40

	Female	3	60

	**ROLE**		

	Clinical mentor	2	40

	Nurse Mentor	3	60


### Themes identified

This study findings yielded 2 main themes: Theme 1: Barriers to integrating mental health services into routine ART services and Theme 2: Enablers to integrating mental health services into routine ART service (Table 2). The enablers and barriers to integration are classified at individual, healthcare system, and community level. Additionally, factors related to stigma and discrimination were identified.

**Table 2:** Themes and subthemes emerged from patient and Healthcare worker perspectives on integration of mental health services into routine ART services in Windhoek, Namibia, 2022.


Barriers integration	**Individual level**

	• Lack of awareness of mental illness among PLHIV

	**Health care system**

	• Negative attitude of healthcare workers

	• Limited space at health facilities

	• Financial resources for staff/shortage of staff

	• Lack of training of HCW

	• Lack of social workers at health facilities

	• Lack of leadership support

	• Fragmented referral system

	• HCW stigma towards PLHIV with mental illness

	**Stigma, fear and discrimination**

	• Fear to discuss mental issues with HCW

	• Fear of discrimination and gossip due to being HIV positive

Enablers integration	**Health Care System**

	• Leadership support

	• Policies, guidelines and SOPs for integration

	• Training curriculum and training of healthcare workers

	• Monitoring, evaluation & reporting systems for mental health services

	• Availability of social workers or psychologists at health facilities

	• Integrated screening for mental illness into routine screening

	• Working hours of health facilities

	**Community Level**

	• Community sensitization and awareness creation among patients to minimize stigma

	• Support groups for mental health in the community

	• IEC materials in local languages


**HCW: Healthcare workers, SOPs: Standard Operating Procedures, PLHIV: People Living with HIV**.

#### Theme 1: Barriers to integrating mental health services

##### Individual related barriers

###### Lack of awareness of mental illness among PLHIV

Participants in the study noted that mental illnesses are prevalent among PLHIV, yet there’s insufficient awareness about these issues within the community. This lack of awareness hinders the reporting of mental health concerns, potentially affecting the utilization of integrated mental health services in routine HIV care. Educating and counseling PLHIV about mental illnesses could enhance their ability to report these issues accurately at healthcare facilities.

*“Like now, a lot have mentally disturbed people have been mentally disturbed, but they can’t come and report that case, but if you educate them, like the way you are counselling people here (at the health facility), that’s how we came in-line, that’s the only way to educate people.” #*Patient 2, 40 yrs, Male

##### Health care system-related barriers

###### Negative attitude of healthcare workers

Several study participants indicated that a negative attitude from healthcare workers towards PLHIV with mental illnesses represents a substantial barrier. They observed that rudeness from healthcare workers makes it difficult for patients to discuss their issues and that the way healthcare workers interact with PLHIV can determine whether patients engage successfully.

*“if the environment (health facility) itself is not fine, if there are two or three people that are rude with people. The people will never come out.”* ~ Patient 4, 26 yrs, M

*“Another barrier is the attitude of us Healthcare workers if we don’t talk to our patients nicely, we will not be able to get anything as the way how we approach our patients ~ HCW (Nurse), 41 yrs, F*

###### Limited space at health facilities

Most respondents reported that inadequate physical space and infrastructure at health facilities hinder integration, as there isn’t enough room to ensure a mental health service-friendly environment. Another respondent highlighted both lack of space and staff as barriers to integration, as limited space makes health facilities unfriendly for PLHIV with mental illness. Furthermore, the lack of space compromises privacy and emphasizes the need for designated mental health areas.

*“Also create mental health-friendly corners that will allow us to have one-on-one sessions with clients in a private space.” HM, 35 years, F*

###### Financial resources for staff/shortage of staff

Concerns about staffing shortages were cited to impact the integration of services, as healthcare workers noted that insufficient staff could lead to time-consuming patient screenings and emphasized that financial constraints hinder hiring additional personnel. These challenges, compounded by existing heavy workloads, pose significant barriers to effectively implementing integrated mental health services into routine HIV care.

*“it would be time-consuming if we provide it (mental health services) to very patient and we won’t manage to see all patients in the queue* ~ HCW (Doctor), 32 yrs, F

*“it can be the experts that need to be employed, which will cost money, and it can take some* time.” HCW (Health Assistant), 28 yrs, M

Some participants reported that human resources for mental health integration is a potential barrier and that a high patient-healthcare worker ratio can affect proper delivery service.

*“The other thing is the patient-to-healthcare worker ratio; you might want to do a proper screening, but looking at the long queue, you might not do justice to this one client.”* Health Manager, 35 yrs, F

###### Lack of training of HCW

Participants reported that lack of training for healthcare workers in mental health services can impede integration. Healthcare workers must receive training on screening for mental illnesses and incorporating mental health services into routine care.

*“If staff are not trained in how to screen patient or how to design or how to integrate we don’t have any package. we need to train our healthcare workers how to screen patients because even if we say let’s do mental health, but people are not trained then we may not really succeed.”* HCW (Nurse mentor), 34 yrs, Female

###### Lack of social workers in health facilities

The need to have social workers and psychologists stationed at health facilities to facilitate easier access to services for patients needing counselling services was highlighted. The lack of these professionals will hinder the successful integration of mental health services.

*“We need social workers readily available in the facility or psychologists to succeed (with integration)” ~ HCW (doctor), 32 yrs, F*

###### Lack of leadership support

Most respondents highlighted the lack of management and leadership support as a significant barrier to integration. They stressed that leadership support is crucial because leaders are responsible for allocating the necessary resources to integrate mental health services effectively and provide oversight to the mental health program.

*“I think the first one is when there is no leadership support, for example there is nothing from the leaders we don’t have enough resources like staff issues like training. I will also touch on leadership support because if leaders don’t have any ideas, they will not even delegate healthcare workers, they will not be able to supervise and they will not be able to oversee the program.” HM, (nurse mentor), 34 years, F*

###### Fragmented referral system

A few respondents indicated that since mental health services are not integrated, the current referral system for mental health services poses a barrier to integration, as healthcare workers often do not know where to refer patients or how to follow up on their care.

*“Our referral system is another challenge; you might come across cases where you don’t know what to do, and the is no straight channel or specific point where you can refer the client and maybe later even get feedback.” ~ Health Manager (NM), 35 yrs*

Inefficiency in the referral process was highlighted, noting the absence of dedicated referral forms for mental health services. As a result, healthcare workers frequently lack clarity on whether patients have accessed required mental healthcare. The respondent indicated that referrals are currently documented solely in the patient’s health passport.

“*we don’t write any referral letter, we just write in their health passport”. HCW, 32 years, F*

###### HCW stigma towards PLHIV with mental illness

Healthcare worker stigmatization of PLHIV with mental illness was reported as a barrier to mental health service integration and service provision.

*“Even healthcare workers, we do stigmatize our clients in the sense that when a mentally ill person walks in seeking help, we don’t really pay attention and will be like this person is mad and why should I waste my time. We focus on other people. So, if the healthcare workers are not trained and sensitized, they will continue with the stigma, that’s another barrier. HCW, 42 yrs, Male*

### Fear, stigma, and discrimination

Fear of disclosing mental illness was the most reported barrier that PLHIV face in addition to their HIV status disclosure. Some respondents mentioned that PLHIV are afraid of being laughed at if they seek mental health services. Additionally, fear of discrimination was cited as a barrier to seeking mental health services.

*“Most of the time, we people who are HIV positive are always discriminated against. You know people like gossiping, name-calling, and exposing others. And just being discriminated” Patient7, 22, F, Single*

#### Theme 2: Enablers to integrating mental health services

##### Health Care System

###### Leadership support

Some participants, particularly health managers, stated that leadership support is crucial and that leaders need to be sensitized to the importance of integration. They emphasized that sensitizing leaders would help establish the necessary policies and provide training for healthcare workers, including the development of appropriate training curriculums for mental health integration.

*“The first one is leadership support; I think we need sensitize our leaders to understand the importance of integration its very vital. is no leadership support for example there is nothing from the leaders we don’t have enough resources like staff issues like training. If staff are not trained how to screen patient or how to design or how to integrate, we don’t have any package” HCW, 34 yrs, M*

###### Policies, guidelines, and SOPs for integration

Healthcare workers and managers stressed the need for treatment guidelines, job aids, and posters on mental health management in ART facilities to support integration. They also highlighted the importance of training healthcare workers in the country’s integration strategy for mental health services in HIV programs. One of the respondents said:

*“I think we need to sensitize our leaders to understand the importance of integration. It’s very vital.”* ~ *Health Manager, 34 yrs, F*

###### Training curriculum and training of healthcare workers

Training healthcare workers, especially in screening and treating mental disorders, is consistently cited as the primary facilitator for integrating mental health services. Additionally, respondents underscored the significance of screening tool development and the provision of ongoing training to ensure successful integration of mental health care.

*“develop the curriculum for training for healthcare workers and then from there we can also train our healthcare workers on how to do this integrations”* ~ HM, 34 yrs, F, NM

###### Monitoring, Evaluation & reporting systems for mental health services

Poor monitoring and evaluation (M&E) systems for mental health services were reported to contribute to gaps in service provision. Some participants noted that when systems for reporting on services are established, it holds healthcare workers accountable for delivering those services. They said:

*The problem with our ministry is, if the is no reporting system, things don’t work. We need to come up with M&E tools that will enable us to account for the numbers* ~ HM, 35 yrs, F

###### Availability of social workers or psychologists at health facilities

The availability of social workers at health facilities to facilitate referrals and linkages for mental health services was reported as an enabler for integration.

“*We need social workers readily available in the facility or psychologists.”* HCW, 32 yrs, F

###### Integrated screening for mental illness into routine screening

Most respondents indicated that regular screening for mental illness among PLHIV by a Healthcare worker is a significant enabler for seeking mental health services. They reported that Healthcare workers are better trained in mental illness identification than patients and knowing that regular screening is available will encourage patients to seek these services.

*“What I can suggest is that, for the doctors to be asking, are you fine? And go into detail like, were you feeling, them (healthcare workers) they know if a person is going through depression, they know what they go through (signs and symptoms.* ~ Patient 4, 26Y, M, Single

###### Working hours of health facilities

Health facility working hours make it a barrier for PLHIV to seek mental health services such as counselling, as the clinics close at 5 p.m., when they are just knocking off at work. They said:

*“If we get like counselling places like where you can go any time of the day that’s open from 7am -7pm, because most of us are working also, if knock off you know, I can go here and explain to these people what illness I am having. So if we get a place like that, it will be very fine* ~ Patient5, 39 yrs, F

##### Community Level Barriers

###### Community sensitization and awareness creation among patients to minimize stigma

Community sensitization was cited as an enabler for integration, emphasizing its importance in addressing the stigma of mental illness among PLHIV. Educating and sensitizing the community are key factors in integrating mental health services into routine HIV programs. Community mobilization is perceived to help reduce stigma and improve case reporting.

“*if they have a mental illness, imagine the stigma they will go through, so if the community is sensitized, at least they will understand and treat them as one and that they are also still people and they need help, so after this, their mentality will change and the stigma will be reduced”*. HCW, 28 yrs, M

###### Support groups for mental health in the community

Establishing mental health support groups in the community is reported to aid integration efforts. These support groups would help raise awareness and provide support to PLHIV with mental illnesses.

*“I think in the community they can introduce support groups, like here at the clinic we identify hand-full patients to form a group to have awareness programs in the community”*. *HCW, 38 yrs, F*

###### IEC materials in local languages

Community mobilization with the use of Information, Education, and Communication (IEC) materials in local or vernacular languages to ensure that community members can effectively use and understand them is reported to facilitate mental health awareness.

“*Hand out pamphlets translated in vernacular languages and posters to create awareness”*. HCW, 28 yrs, M

## Discussion

This paper aimed to explore healthcare workers’ and patients’ opinions on enablers and barriers to integrating mental health services in routine HIV care in Windhoek, Namibia. A phenomenological approach was employed to explore the perspectives of PLHIV, healthcare providers delivering ART services, and health managers supporting the national HIV program.

Understanding these perspectives is valuable for successfully integrating mental health services into routine HIV programs. In this study, barriers at individual and healthcare system levels were identified, while enablers at healthcare system and community-level factors were highlighted.

Various research studies have reported the role that leadership plays in either being an enabler or a barrier to integration [42,43,44,45] which is consistent with our study findings that report that leadership is a key enabler and likewise a barrier to integration. Our study findings indicate that effective leadership and management are crucial in promoting integration. The findings from this study further imply that effective integration heavily depends on the endorsement and commitment of healthcare leadership. Leaders and managers play a vital role in allocating resources for staff training [46], mental health services [47], infrastructure improvement, and the creation of essential policies and guidelines that facilitate integration [46,48]. By prioritizing these aspects, leaders and managers ensure that the necessary support systems are in place to foster a cohesive and well-integrated health system. Also, our study findings suggest the importance of sensitizing and educating policy makers on the importance of integration of mental health services to facilitate successful integration. These findings align with a cross-sectional study conducted in a health facility in Jimma Zone, in Ethiopia, among primary healthcare workers, which also emphasized the importance of leadership and management support in integration [43].

Furthermore, the cross-sectional study in Jimma Zone, Ethiopia, highlighted a significant barrier: the limited understanding of mental health integration among health managers. Several key obstacles, including the lack of government capacity, readiness, and prioritization of screening and managing perinatal depression, as well as the absence of mental health policies and strategies, were identified in the Ethiopian study.

Findings from this study show that integration efforts should be complemented by detailed guidelines, standard operating procedures (SOPs), job aids, and comprehensive training for healthcare staff. These findings are consistent with a systematic review that assessed enablers and barriers to integrating maternal health services into antenatal care in low- and middle-income countries [49]. The systematic review findings report that comprehensive training and well-defined care guidelines are crucial for equipping individuals with the necessary knowledge and skills to deliver integrated services effectively [49]. Also, without guidelines, there is a chance that services will be provided better than they should [49].

Training of healthcare workers is key for successful integration, and lack of it is a barrier. Our study findings report that healthcare workers and managers believe that successful integration will require the training of healthcare workers on mental illness screening, treatment, and management. Empirical evidence underscores that training of healthcare workers for competency is key because even if people did access health centers, Healthcare workers often lacked the skills to identify and record patients with mental illnesses [50]. These findings are in line with opinions expressed by healthcare workers in some studies conducted in Ethiopia [43,51], whereby a health facility-based cross-sectional study conducted in Jimma zone among health care providers indicates that health care providers reported a need for further training to competently deliver mental health services [43]. Additionally, the qualitative interview conducted among health administrators among Ministry of Health staff in the Amhara regional state of Ethiopia also supports that the training of healthcare workers is key in delivering integrated mental health services as healthcare workers have expressed their incompetency in screening patients for mental illness [51].

Some studies have highlighted that service users often lack essential knowledge about the appropriate interventions needed to prevent and promote mental health [52]. This gap in understanding extends to awareness of where to seek biomedical treatment for mental health issues [52]. Many individuals are unaware of the available resources, treatments, and preventative measures that could significantly improve their mental well-being. This lack of knowledge can result in delayed or inadequate care, worsening mental health conditions, and missed opportunities for early intervention and prevention. Our findings show that PLHIV have identified a lack of mental health awareness as a substantial barrier in integration. Therefore, the lack of relevant information on mental health services that exist is a hindrance to users (PLHIV) from seeking needed services. This phenomenon is supported by findings from a qualitative study conducted in Ghana, Uganda, Zambia, and South Africa, which aimed to develop and evaluate mental health policy interventions in the four countries. The study was conducted among national and regional stakeholders in these countries, and the findings indicated that mental health has received low priority not only among decision makers but also among the communities [50]. The invisibility of people with mental illness in the community is believed to contribute to the poor understanding and appreciation of the prevalence of mental illness [50,53]. When individuals with mental health conditions are not visible or openly acknowledged, it leads to a lack of awareness and recognition of how widespread these issues are within the community. This invisibility can perpetuate stigma, reduce empathy, and hinder efforts to provide adequate support and resources for those affected, but it also results in people hiding mental illness to avoid stigma [50]. Increasing the visibility and awareness of mental health issues is essential to improving community appreciation of their prevalence and fostering a more supportive and inclusive environment. Since stigma, fear, and discrimination of PLHIV have been identified as obstacles to successful integration by other literature, our study’s findings are consistent with these conclusions.

Mental illness-related stigma is not only experienced by PLHIV; some studies have also reported stigma experienced by healthcare workers. A qualitative study conducted among healthcare workers in three public urban HIV treatment facilities in Yaoundé, Cameroon, has reported that healthcare workers experienced stigma as they did not want to partner or work at a mental health unit for fear that others also assume that they have mental illnesses too [53]. Additionally, both groups expressed concern that others would assume they had mental disorders simply because of their physical proximity to or collaboration with specialized mental health providers [53]. Stigma can hinder collaboration and care, as individuals may avoid seeking help or working with mental health professionals out of fear of judgment or misunderstanding. While our study did not find reports of healthcare worker stigma, it’s crucial to consider its potential impact when integrating mental health services into HIV care. Such stigma can greatly affect patient care and support, potentially worsening health outcomes and marginalizing those with HIV.

Our study underscores the importance of having dedicated infrastructure and space to facilitate the integration of mental health services into routine HIV care. Sufficient space is key in ensuring confidentiality and openness of PLHIV seeking mental health services [53]. The importance of sufficient space at health facilities is also commonly reported in other studies focusing on mental health integration in HIV clinic settings [52,53,54]. This concern is aligned with the findings of this study, where lack of space was reported as a factor contributing to an “unfriendly” environment for mental health integration for both PLHIV and healthcare workers.

## Implications of study findings

The outcome of this study has broad implications for integrating mental health services into primary health care settings, especially for people living with PLHIV in Namibia. The rise of mental illness among PLHIV necessitates strengthening the primary health care system to address this need. This study is crucial in identifying barriers and facilitators of integrating mental health services into routine HIV care from the perspectives of patients, healthcare workers, and health managers. These findings contribute valuable insights into the perceived barriers and facilitators, enhancing the knowledge base for effectively addressing the mental health needs of PLHIV.

## Limitations

Qualitative methodologies provide data that may only apply within the study’s specific context. Interviews and observations offer rich insights into individual experiences but are often not generalizable to wider populations. This study’s participants were exclusively from outpatient ART facilities, excluding patients who did not seek treatment at these clinics and those who were not comfortable expressing themselves in English. While focusing on a single ART facility provides detailed insights, it limits representativeness. Perspectives of individuals seeking treatment elsewhere or healthcare workers who do not present during interviews might not be captured. Caution is needed when generalizing these findings to broader populations or healthcare settings.

## Conclusions

Understanding the experiences and perceptions of PLHIV and ART healthcare workers is crucial for designing effective strategies to address rising mental health issues among PLHIV through the integration of mental health services. Individual-level barriers include limited awareness of mental illnesses among PLHIV, while healthcare system-related barriers encompass healthcare worker attitudes, space constraints at health facilities, financial limitations for staffing, inadequate training, lack of leadership support, and stigma toward PLHIV by healthcare workers. Addressing these barriers holistically can enhance health outcomes for PLHIV by preventing and reducing the prevalence of mental illnesses in this vulnerable population.
